# Light Emitting Spin Active Electronic States in Ultra-Thin Mn Doped CdSe Layered Nanosheets

**DOI:** 10.1038/s41598-019-38974-0

**Published:** 2019-02-12

**Authors:** O. Halder, B. Satpati, P. Rajput, N. Mohapatra, S. N. Jha, J. Suffczyński, W. Pacuski, S. Rath

**Affiliations:** 1 School of Basic Sciences, Indian Institute of Technology Bhubaneswar, Jatni, 752 050 Khurda, India; 2 Saha Institute of Nuclear Physics, 1/AF Bidhannagar, Kolkata, 700 064 India; 3 Atomic & Molecular Physics Division, Bhabha Atomic Research Centre, Trombay, Mumbai, 400085 India; 4 Institute of Experimental Physics, Faculty of Physics, University of Warsaw, Pasteura 5 St., Warsaw, 02-093 Poland

## Abstract

The layered nanosheets exhibit a variety of physical and optical properties originating from amalgamation of intra- and inter- layer electronic interactions, which makes them promising materials for advanced devices with varsatile controlling channels. In particular, the dilute magnetic semiconductor multilayered nanosheets have promising optical, electrical and magnetic properties that have been less explored so far. Here, the spin permissible optical properties from solvothermally grown Mn doped CdSe (thickness ~2.26 nm) multilayered nanosheets are reported on. The presence of multi-phase magnetic orderings with a sharp ferromagnetic transition at temperature ~48 K pertinent to the stabilization and co-existence of Mn^2+^ and Mn^3+^ based local phases have been observed from the (Cd,Mn)Se layered nanosheets corroborating to the x-ray absorption near edge structure, electron paramagnetic resonance, Raman scattering and magnetic measurements. The optical absorption and photoluminescence (PL) studies at room temperature affirm wide array of optical properties in the visible regime corresponding to the band edge and intriguing dopant-phase mediated spin approved transitions. The circularly polarized magneto-PL and life time analysis exhibits the spin-polarized fast radiative transitions confirming the presence of spin-active electronic states.

## Introduction

The two-dimensional (2D) semiconductors^1^ and doping effect in its layered structures are an active domain of research to offer advanced optical^2^, conductive^3^ and magnetic properties^4,5^ as a prospect of new generation device applications. Simultaneously, no effective methods of enhancing the sensitivity of these materials with reference to the external field has been established so far. Skillful doping of transition metal ions in nanostructures alter their spin degrees of freedom with promising giant Zeeman splitting^3,6^, magneto-optical^7^ and fast spin-lattice dynamics^8^. For instance, it activates a long range magnetic ordering crucial for spin-photonics^9^, spintronics^10^ and carrier-induced magnetism^5^. Moreover, the reports on doping manganese (Mn) ions are currently establishing the coexsistance of Mn^2+^/Mn^3+^ states leading to various interactions and spin-allowed transitions^11^. In particular to Mn doped bulk cadmium selenide ((Cd,Mn)Se), antiferromagnetism is stereotype^1,12,13^ due to the short range *d–d* interactions. However, the calculations predict hole-induced long range ferromagnetism (FM) from nanocluster state or co-doping^14,15^. Besides this, M. Sawicki *et al*.^16^, G. L. Gutsev *et al*.^14,17,18^ and J. Yang^19^ expressed intriguing magnetic states and related spin-active optical properties depending upon the concentration and ionic co-ordinations of the dopant. Similarly, the optical studies of a very low Mn doped CdSe/ZnSe core-shell quantum dots^20,21^ predicts insignificant role of an individual Mn^2+^ on the properties of host lattice. Nevertheless, studies on different quantum structures report on quenchning and shifting^22^ of optical properties with the Mn^2+^ state. Despite of the low solubility, several groups have initiated the Mn doping in various structure and composition^23–25^ regardless of the ordinary properties. However, the concerns over doping stabilization in multilayered nanostuctures and its correlated properties orginating from intra-layer and inter-layer connected electronic states are yet to be explored.

In this work, we report on (i) the successful doping of Mn into the cadmium selenide (CdSe) multilayered nanosheets (LNSs) lattice using a solvothermal route, (ii) strain induced stabilization and co-exitance of Mn^2+^/Mn^3+^ states leading to multi-phase magnetic ordering with sharp ferromagnetic ordering at temperature ~48 K, (iii) spin-polarized excitonic properties and (iv) fast spin permitted electronic transitions covering the visible realm at room temperature.

## Synthesis Method

Mn doped CdSe LNSs were synthesized via surfactant assisted solvothermal growth technique using cadmium chloride (CdCl_2_), selenium (Se) powder and manganese chloride (MnCl_2_) as the source materials and octylamine as a surfactant. For the synthesis of (Cd,Mn)Se LNSs, the typical parameters comprising a 0.2750 g of CdCl_2_ and 0.0013 g of MnCl_2_ were mixed in a glass container containing 10 ml of octylamine under continuous stirring followed by the constant heating at 393 K for 2 hrs to achieve the Cd-Mn – octylamine complex. Similarly, a 0.0513 g of selenium (Se) powder was added to the 10 ml of octylamine under vigorous stirring followed by the addition of CdMn-octylamine complex at constant temperature, 423 K for 24 hrs aiming the growth of the (Cd,Mn)Se LNSs. Then, the (Cd,Mn)Se LNSs were extracted by repeated washing with trioctylphosphine and ethanol.

## Experimental

The samples were characterized by transmission electron microscopy (TEM) using FEI, TF30 set up operated at 300 kV equipped with a GATAN Orius CCD camera and energy dispersive x-ray (EDX) facility as an attachment was used for the TEM analysis of the samples. For the TEM studies, samples were collected on the carbon coated copper grid. The X-ray absorption near edge structures (XANES) measurements at Magnanese K-edge were carried out at BL-9, Scanning EXAFS Beamline of Indus-2, RRCAT Indore, India. The measurements were done in a fluorescence mode using Vortex energy dispersive detector. The beamline consists of Rh/Pt coated meridional cylindrical mirror for collimation and a Si (111) double crystal monochromator (DCM) to select excitation energy of Mn K-edge (6539 eV). The second crystal of the DCM is a saggital cylinder which provides beam focused in horizontal direction. The inferences in the energy range -20 eV below to +20 eV above the Mn K-edge were obtained by utilizing the linear combination fitting (LCF) protocol. The vibrational properties of the samples were analyzed using Horiba-T64000 micro-Raman spectrometer coupled with Peltier cooled CCD detector having spectral resolution as 0.8 cm^−1^ in presence of a laser excitation of wavelength, 488 nm and power, 20 mW from Argon-ion source focused to a 5 µm circular spot. The Raman spectra were collected in a back-scattering geometry. The magnetic measurements of the (Cd,Mn)Se LNSs powdered sample were carried out using Quantum design make physical properties measurement system (PPMS). Room temperature based optical absorption measurements were done by dispersed in a chloroform using a UV-1800 UV-VIS Shimadzu spectrophotometer. The background corrections were made using chloroform as the reference sample. For the magneto-photoluminescence (M-PL) spectroscopy, experimental conditions are the following: the M-PL is excited with a continuous wave 405 nm (3.06 eV) laser. The diameter of the laser spot on the sample surface is 0.1 mm and the excitation power density is of around 5 W/cm^2^. The sample is immersed in superfluid helium at temperature of ~2 K. The PL measurements are performed in the Faraday configuration (***B***||***k***) in a cryostat equipped with a superconducting coil. The coil provides a magnetic field of up to 10 T. A Peltier-cooled CCD camera coupled to a grating (1200 grooves/mm) monochromator serves as a detector (overall spectral resolution of the setup 0.12 meV). A long wavelength pass filter placed at the entrance of the monochromator cuts off the stray light of the laser. The x-ray powder diffraction (XRD) measurements were carried out using the Bruker D8 ADVANCE instrument. The electron paramagnetic resonance (EPR) studies were performed using Bruker EMX PLus [ER 073], EPR instrument at low temperature regulated by continuous flow of liquid nitrogen. Here, the instrument optimization has been achieved at magnetic filed, 3 Tesla and frequency, 9.47 GHz. The time resolve photoluminescence were achieved using a Horiba scientific system coupled with MCP-Hamamatsu photomultiplier detector, having time resolution 5 ps. An excitation laser source of wavelength, 379 nm and pulse rate ~200 ps was used for the measurements.

## Results and Discussion

**Figure 1:**
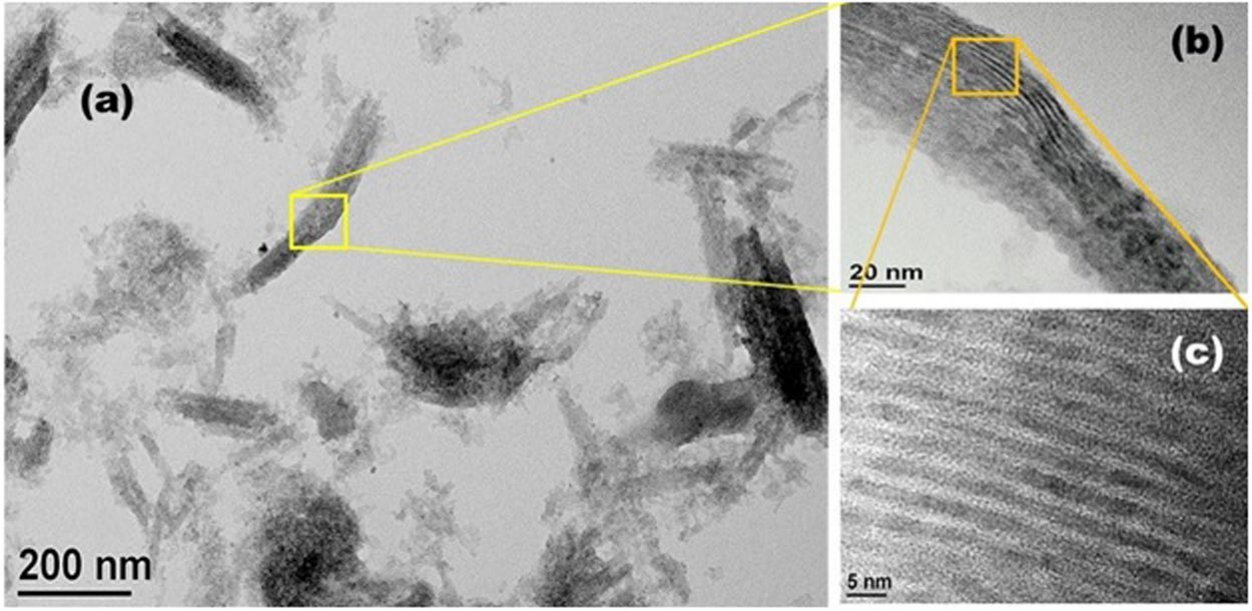
The TEM micrograph of (**a**) Mn doped CdSe nanosheets, (**b**) the zoomed region marked by yellow box revealing the layered nanosheets, and (**c**) high resolution TEM of the zoomed region marked by orange box. The thickness of the indivisual nanosheets is estimated as 2.26 nm.

In order to understand the doping and nanolayer structure formation, the samples were characterized by the TEM and EDX measurements. The images shown in Fig. 1(a) confirm the growth of ultra-thin nanosheets with average length and width of 155 ± 20 nm and 40 ± 5 nm, respectively. The yellow box area is the side view of the NS, the magnified image is shown in Fig. 1(b). It clearly shows the stackings and presence of layer structure in the NSs. Further, the high resolution TEM (HRTEM) image from the organge box area shown in Fig. 1(c) provides a clear view of stacked nanolayers with average thickness of the individual nanolayer as 2.26 nm. The average gap between two consecutive nanolayers is found to be 1.28 nm. The lattice distance is estimated to be 3.3 Å corresponding to the hexagonal phase of the CdSe.

**Figure 2:**
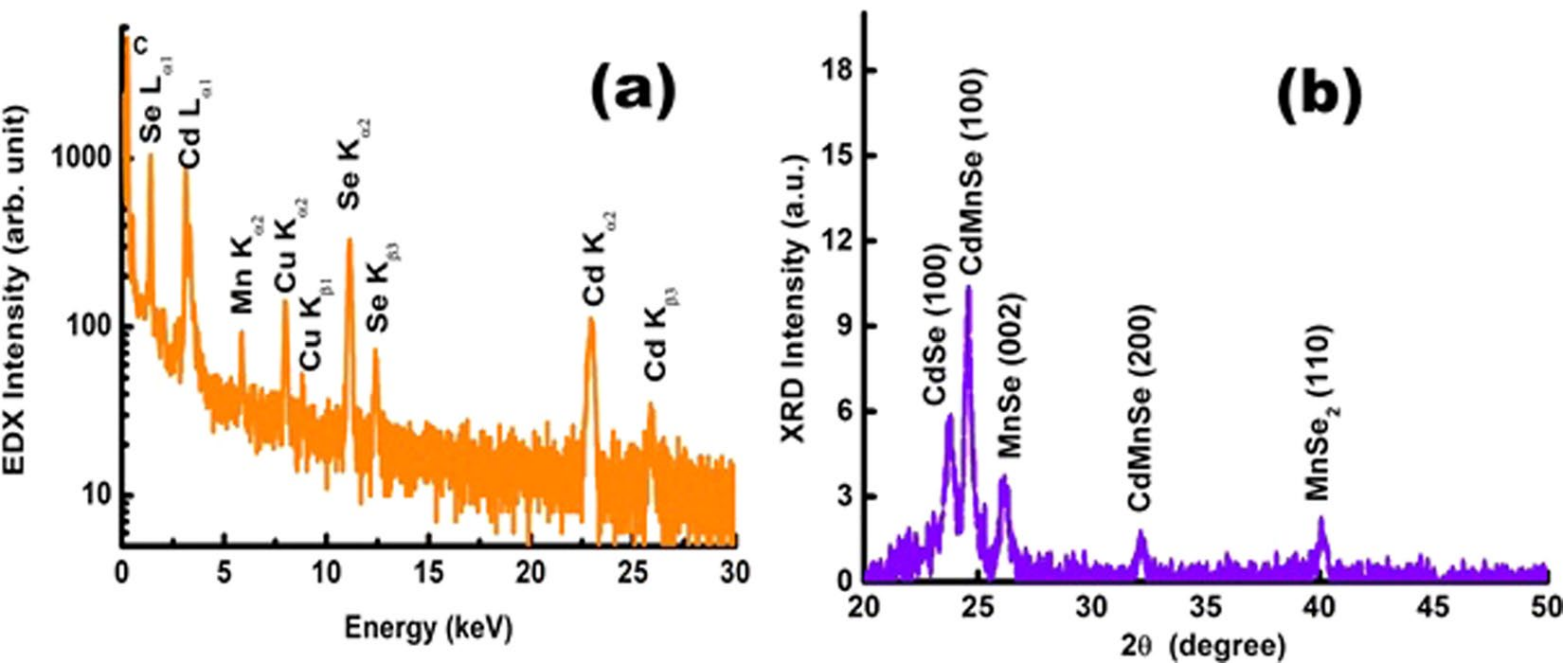
The EDX spectrum (**a**) and XRD spectrum (**b**) of the (Cd,Mn)Se LNS.

The composition of the samples were confirmed from EDX measurement and the spectrum is shown in Fig. 2(a). From the EDX spectral analysis, the atomic percentage of the Cd, Se and Mn in the LNSs are estimated to be 45.23%, 49.35% and 5.40% respectively referring to the composition of Cd_0.9_Mn_0.1_Se. The additional copper (Cu) - and carbon - line observed in the EDX may be from the carbon coated Cu grid as a substrate.

The crystallinity of the samples was verified using the XRD measurements. The XRD spectrum shown in Fig. 2(b) consists of crystallographic planes peaking at 2θ (in degree) as 23.80, 24.60, 25.29, 26.16, 32.14, 40.07 respectively. Comparing with standard data (JCPDS file # 77-2307), the 2θ peaks at 23.80 and 25.29 may be attributed to the hexagonal phase (100) and (002) plane of the CdSe. Moreover, as reported by R. Sharma *et al*.^26^, the 2θ peaks at 24.9 and 26.7 denotes to the (100) and(002) plane of the hexagonal MnSe; and the peak at 32.5 orginating from (200) plane represents the cubic (Cd,Mn)Se like phase. Therefore, in our case, the diffraction 2θ peaks appearing at 24.60 and 26.17, and 32.14 may be the hexagonal and cubic phase of the MnSe respectively. The diffraction line appearing at 40.07 may be attributed to the MnSe_2_ like phase^27^. The lattice parameters, “*a*” (=*b*) and “*c*” of hexagonal phase MnSe present in our sample, have been estimated as 4.172 Å and 6.799 Å respectively. Interestingly, the *c/a* ratio is found to be 1.63 which is higher in comparison to the bulk MnSe (1.61). This eliminates the probability of acquiring individual Mn at the surface as a separate alloy like phase. Rather, it endorses the substitutional nature of the Mn ion along the c-axis of the hexagonal CdSe lattice. Notably, the above crystallographic planes are observed to be broaden and shifted towards lower 2θ. This could be due to the lattice strain refered to quantum structures and doping effect^5,8,28^. The strain has been calculated from^26^ $\beta =\frac{K\,\lambda }{d\,Cos\theta }+\eta \,{\tan }\,\theta$ where, *K* (for 2D lattice) = 0.89, x-ray wavelength, λ = 1.54 Å, *β* = full width of half maxima of difraction peak; resulting the strain, *η* = 1.078%; and size as *d* = 46.84 nm.

**Figure 3:**
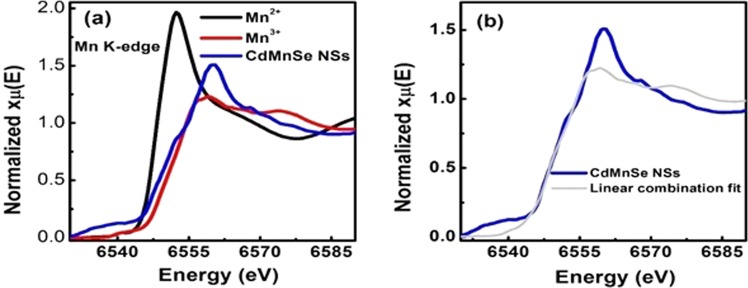
The Mn K-edge XANES spectra of (**a**) (Cd,Mn)Se LNSs, MnO (Mn^2+^) and Mn_2_O_3_ (Mn^3+^) shown by blue, black and red color curve respectively. Here, MnO and Mn_2_O_3_ are the standard samples. (**b**) The grey color curve is the theoretical fit with experimental Mn K-edge XANES data (blue color) using a linear combination protocol in Athena software.

To infer the valence state of the Mn dopant, the XANES has been carried out. Figure 3(a) shows the Mn K-edge XANES spectra of (Cd,Mn)Se LNSs along with the Mn^2+^ and Mn^3+^ as standard references represented by blue, black and red spectrum respectively. Using the linear combination fitting (LCF) from Athena software^29^ in the energy range 6540–6555 eV, the experimental curve is fitted and is shown in Fig. 3(b). The Mn K-edge analysis reveals the coexistence of Mn^2+^ and Mn^3+^ states in the sample. Even though  the Mn^3+^/^2+^ states are witnessed in Mn doped semiconductors^30,31^, Mn^2+^ state is frequently reported (Mn ion substituting Cd and having Se neighbors) for Mn doped CdSe. Therefore, the observation of Mn^3+^ state in our case is exceptionally possible due to the favourable site specific Mn ion substitution by single or double or mixed Cd and Se neighbors interacting within the ultrathin 2D lattice.

**Figure 4:**
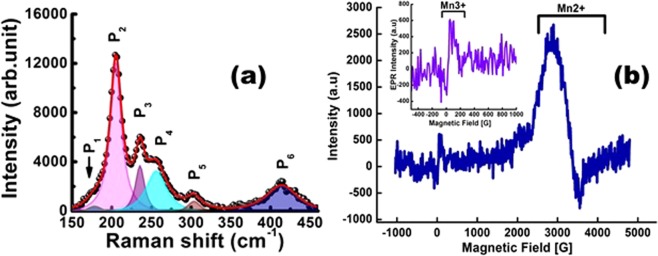
(**a**) The Raman spectrum of the (Cd,Mn)Se NSs at the room temperature. The deconvoluted Gaussian peaks corresponding to the optical phonon modes are described by filling different colors. (**b**) The electron paramagnetic resonance (EPR) spectrum of (Cd,Mn)Se LNSs at tempeature, 100 K. Inset is the zoomed EPR spectrum in the magnetic field range −400–1000 G.

The valence states of the dopant were further assessed from the vibrational properties of the samples using laser Raman scattering measurements shown in Fig. 4(a). The (Cd,Mn)Se LNSs displays a series of Raman modes detected at 178.3 cm^−1^ (P_1_), 205.2 cm^−1^ (P_2_), 235.6 cm^−1^ (P_3_), 256.2 cm^−1^ (P_4_), 303.8 cm^−1^ (P_5_) and 414.3 cm^−1^ (P_6_). The most intense lines, P_2_ and P_6_ are attributed to the first order (1LO) and second order (2LO) longitudinal optical phonon modes of the hexagonal CdSe^32^ phase. The 1LO mode with full-width-half maxima (FWHM) of 17.5 cm^−1^ does not show a phonon confinement effect in contrast to the blue shift (~ 3.3 cm^−1^) observed in 2LO line with FWHM of 49.5 cm^−1^ with respect to the bulk (1LO ~ 205.5 cm^−1^ and 2LO~411 cm^−1^). The peak position of transverse optical (TO) phonon at P_1_ with FWHM as 26.0 cm^−1^ is observed to be blue-shifted by 6.8 cm^−1^ from the bulk (TO ~ 171.5 cm^−1^)^26^. As a result, the TO line is responsible for the lattice vibration along c-axis of wurtzite CdSe phase^33^. The blue-shift in P_1_ indicates a strong compressive strain along c-axis and can be estimated quantitatively using the relation^26^, $\frac{{\rm{\Delta }}\omega }{\omega }={(1+3\frac{{\rm{\Delta }}c}{c})}^{\gamma }-1$; where, $\frac{{\rm{\Delta }}\omega }{\omega }$ is strain induced blue-shift of the TO line in comparison with bulk crystal, $\varepsilon =\frac{{\rm{\Delta }}c}{c}$ is the strain along c-axis and γ is the Gruneisem parameter (γ = 1.1 for CdSe). Using the above relation, the strain, ε is estimated to be 1.2% for (Cd,Mn)Se LNS. Further the peaks, at P_4_ and P_5_ are signified as the phonon modes of the Manganese Selenide (MnSe) like – zinc-blend (ZB)^1,34^ and wurtzite (WZ)^35^ phase respectively. This attributes, defines the presence of different phases to the multivalency of the Mn ions. The peak at P_3_ may be arising from the V_Cd_ – V_Mn_ – V_Se_ like states^28,36^.

Since the Raman intensity of CdSe-like and MnSe-like modes are strongly dependent on the molar fraction of the Mn dopant, the average molar concentration of Mn can be estimated quantitatively using the expression^37^ $x=\frac{{I}_{MnSe}}{{I}_{CdSe}+{I}_{MnSe}}$; where *I*_*CdSe*_ and *I*_*MnSe*_ are the integrated scattering intensities of the CdSe-like and MnSe-like phonons of Cd_0.9_Mn_0.1_Se (calculated from EDX) crystal. Including the correction for Rayleigh factor and thermal occupation factor, the integrated scattering intensity has been evaluated using the expression^38^, $I=\frac{{I}_{m}}{\omega }{({\omega }_{L}-\omega )}^{4}{(n+1)}^{-1}$ where *I*_*m*_ is the measured peak intensity, *ω* is the Raman frequency shift, *ω*_*L*_ is the excitation laser frequency and phonon distribution, $n(\omega )={[\exp (\hslash \omega /{k}_{\beta }T)-1]}^{-1}$. Considering the ZB phase and WZ phase phonon modes of MnSe, the molar fraction of the Mn is estimated to be 0.198 and 0.0583 respectively. This indicates that out of the total Mn content in the LNSs, 77.25% of Mn exhibits ZB-like phase with Se and 22.75% of Mn exhibits WZ-like phase with Se. This can be visualized by considering the Mn doping mechanism in the CdSe clusters observed by G. L. Gutsev *et al*.^18^. As the ionic radius of the Mn is smaller than Cd, the probability of substitution phenomena by Mn dopants may follow- (1) single substitution of Cd ion, (2) double substitution of Cd ions or (3) double substitution of Cd and Se ions by two Mn ions. Since, the affinity of Mn to substitute the Cd ion is stronger, so under condition (2), Mn dimers may have formed by replacing Cd ions along the c-axis (c-c site) or, from the center to the hexagonal surface (c-h site) corresponding to the octa-coordinate or tetra-coordinate respectively. The absence of the pre-edge Mn K-line corresponding to the tetra-coordinate in our XANES spectra rather confirms the octa-cordination environment^39^. Following this, the MnSe clusters of different phases are randomly distributed through out the 2D-crystal to stabilize the lattice^1^.

The local spin environment of the (Cd,Mn)Se LNSs was determined using EPR measurements at temperture 100 K. The EPR spectrum shown in Fig. 4(b) contains two distinct spin splitting states in the magnetic field range of 0–200 G and 2000–4000 G, corresponding to the effective Lande-g factor (*g*_*eff*_) as 41.23 and 2.04 respectively. In general, the *g*_*eff*_ = 2.0 resonance line with well resolved EPR hyperfine structure (*hfs*) is the characterisitcs for the isolated Mn^2+^ ions. The broad and featureless spectrum at *g*_*eff*_ = 2.04 observed in our sample is attributed to the dipolar interaction mediated superimpose of the *hfs* lines revealing the structural modification in Mn^2+^ vicinity. Furthermore, as per D. V. Azamat *et al*.^11^, the Mn^3+^ site is stabilized by an elongated octahedral environment due to the axial strain which produce a static Jahn-Tellar distortion, even at room temperature in the oxide materials, yielding the EPR signal in the magnetic field range of 0–200 G. Therefore, the observation of small EPR signal in our sample at magnetic field, 163.55 Gauss with g_eff_ = 41.23 may have originated from the Mn^3+^ site compensated by an elongated octahedral symmetry. The observed g_eff_ is much higher than in case of the Mn doped CdSe quantum dots^40^ and quantum well^41^ making our sample attractive for spintronic applicaions. Notably, the intensity of EPR corresponding to the Mn^2+^ site is much stronger than to Mn^3+^ site which suggests that the molar concentration of Mn^2+^ dominates over Mn^3+^.

**Figure 5:**
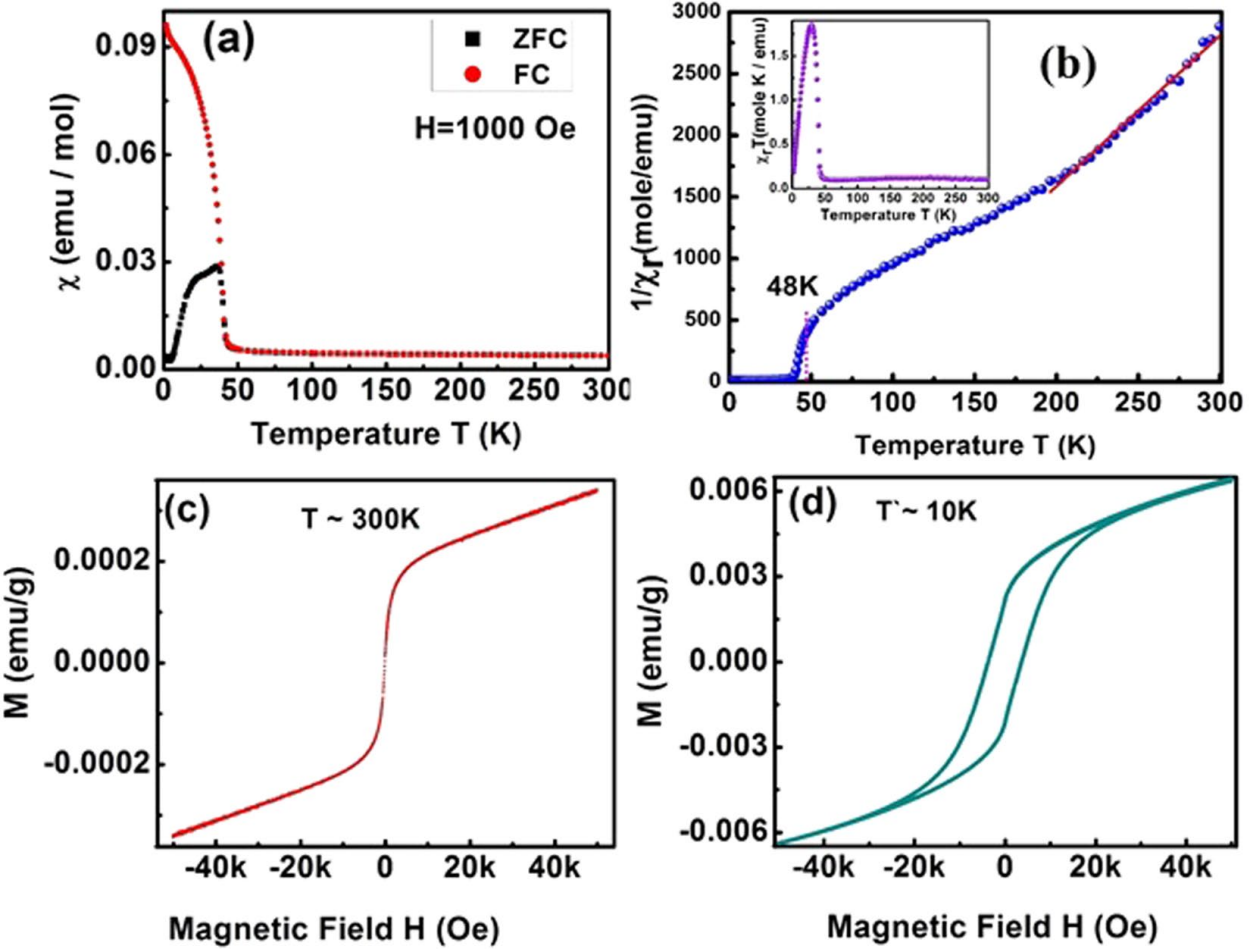
(**a**) The χ versus temperature, T curve in the zero-field-cooled (ZFC) condition indicated by black color filled boxes and field-cooled (FC) condition at magnetic field 1000 Oe indicated by red color filled circles. (**b**) The 1/χ_r_ versus T plot indicated by blue spheres. Inset is the χ_r_T versus T curve indicated by purple color spheres. (**c**) and (**d**) are the M ~H curve at temperature 300 K and 10 K respectively.

The impact of Mn multivalency on the physical properties of the (Cd,Mn)Se was observed through magnetic measurements using Quantum design Dynacool VSM (vibrating sample magnetometer) set up. The magnetic susceptibility (*χ*) of the samples were studied in the temperature range from 2–300 K under zero-field cooled (ZFC) and field cooled (FC) condition of the specimen at a constant magnetic field, 1000 Oe. As observed from Fig. 5(a), the ZFC and FC curves increase concurrently with decrement in the temperature from 300 K followed by a sudden rise in the magnitude at ~48 K. This signifies a ferromagnetic ordering, although ferrimagnetic ordering cannot be ignored. Upon cooling below 48 K, the observed ZFC curve diverges from the FC curve upto a blocking temperature at ~23 K, corresponding to the superparamagnetic behavior^42^. In contrast, the FC curve continues to rise up to the saturation value^14,18,43^. This indicates the presence of strong magnetic anisotropy related to a shape – or to a single ion – (Mn^3+^ configuration) anisotropy, which is super sensitive to strain. In order to understand the magnetic interactions, we analyzed the inverse of the susceptibility versus temperature plot shown in Fig. 5(b) at high temperature (200–300 K) regime. We found a quite standard behavior – diminution of the magnetization with temperature, i.e., a characteristic of systems dominated by superparamagnetism^36,42,44^. Likewise, from the *χ*_*r*_ T versus T plot as shown in the inset of Fig. 5(b), the curve decreases continuously upon decreasing the temperature upto 48 K followed by a peak bound rising till 30 K revealing the presence of ferrimagnetic^5,14,45^ ordering. To reassert the magnetic ordering, the magnetization (M) versus magnetic field (H) measurements was performed. The M~H curve shown in Fig. 5(c) measured at 300 K varies linearly with low magnetic field upto 5000 Oe. This may indicate a presence of high temperature superparamagnetic ordering^5,18,28^ of weakly dispersed small clusters (with ferromagnetic domains), addition to the earlier observed properties. In contrary, the M~H curve at 10 K in Fig. 5(d), reveals a hysteresis loop with remanence as 3.0 milli-emu/g and coercivity of 3682 Oe. These observed values are much higher than that of the superparamagnetic samples suggesting ferromagnetism at low temperatures. Therefore, the two phases of magnetic ordering noticeable in our samples are – the one below critical temperature ~48 K, and the other above the critical temperature to room temperature. This approves the proficiency of our sample for both superparamagnetic and ferromagnetic applications.

**Figure 6:**
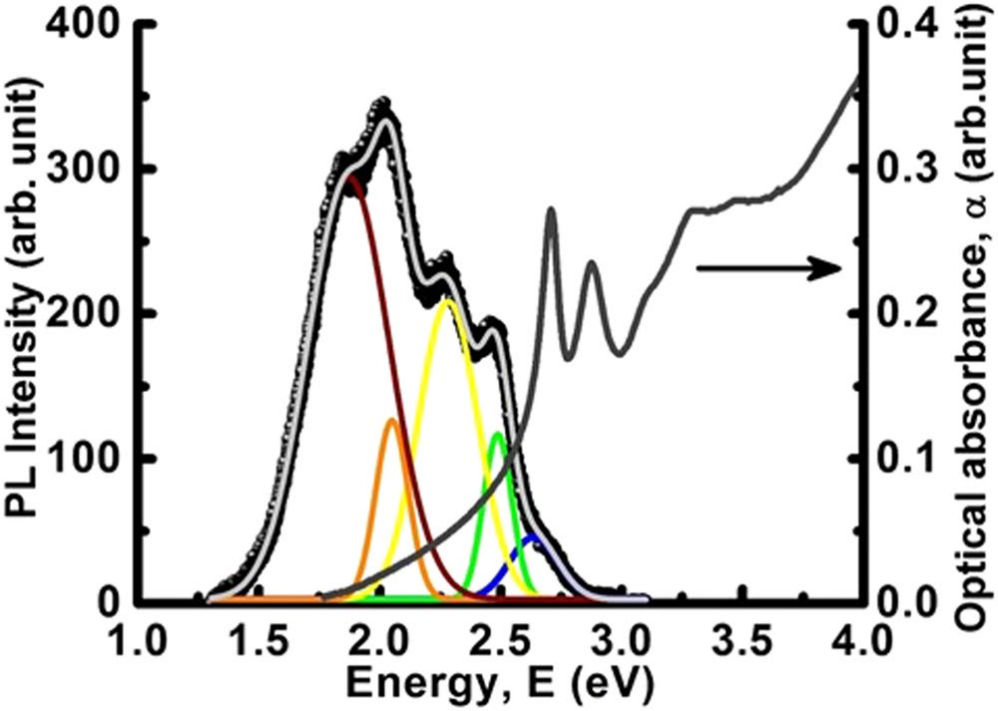
The optical absorption spectrum (indicated by right arrow) and photoluminescence spectrum of Mn doped CdSe nanosheets. The colored spectra are the fitted lines using Gaussian function indicating different transitions.

The spin degrees of freedom triggered by the Mn doping on the electronic structure of the CdSe LNSs was examined by room temperature optical absorption and photoluminescence measurements. The absorption spectrum of the LNSs marked by the right arrow in Fig. 6 with strong excitonic peaks at 2.72 eV and 2.87 eV corresponds to the optical transitions between heavy hole (1hh) and light hole (1lh) to conduction (1e) band respectively. In comparison to the CdSe LNSs^2^ (1hh – 1e ~ 2.73 eV and 1lh – 1e ~2.90 eV), the excitonic peaks corresponding to the transitions 1hh – 1e and 1lh – 1e appears to be red-shifted by 30 meV and 20 meV respectively in the (Cd,Mn)Se LNSs with a long tailing towards the low energy. Whereas, the PL spectrum in Fig. 6 covers a wide visible range, peaking at the energies 2.64 eV, 2.48 eV, 2.28 eV, 2.05 eV and 1.87 eV, respectively. The emission line at 2.64 eV attributes to the near band edge emission (NBE) with a Stoke’s shift of 90 meV in reference to the absorption line. According to the electronic structure of Mn^2+^ (five localized electrons in the *d-*orbital)^8,41,46^ inside the host, the *d-*orbital splits into higher ^4^*T*_2_, ^4^*T*_1_ states to the ground ^6^*A*_1_ state with a splitting gap of 2.50 eV and 2.12 eV^47,48^. Therefore, the emission lines appear at 2.48 eV and 2.05 eV may be attributed to the ^4^*T*_2_ → ^6^*A*_1_ and ^4^*T*_1_ → ^6^*A*_1_ transitions respectively. Again, the substitution of Cd by Mn ion creates deep trap states originating from either a single or double substitution. Interestingly, the double substitution vacancy like, *V*_*Cd*_ *− V*_*Se*_ is observed to be more stable due to the high activation energy. As a result, the carriers are localized at Se – ligand around the *V*_*C*d_ vacancy yielding deep trap states around at 2.25 eV and 1.75 eV corresponding to the *c–c* axial site and *c-h* basal site substitution^1,20,48^. Thus, in our case, the bands observed at 2.28 eV and 1.87 eV may be assigned to the deep trap states originating from the formation of divacancy structures. From the full width of half maxima (FWHM) analysis of the deep trap states, the Huang-Rhys^49^ factor “*S*” representating the strength of electron-phonon coupling $({\rm{\Delta }}(FWHM)=\hslash {\omega }_{LO}2\sqrt{S}$ where ħω_LO_ = 26 meV is the LO-phonon energy of bulk CdSe) has been calculated as 22 and 42 respectively. The Stoke’s shift and high coupling constant display an inherent participation of the Mn electrons to maintain the stability of the crystal^50^. Moreover, if the Mn^3+^ (four localized *d* electrons) acts as non-codopant, it produces lattice strain (seen from the XRD and Raman analysis). Under this environment, the *d-*orbital undergoes a Jahn-Teller effect and splits into excited ^5^*E* state to the ground ^5^*T*_2_ states with gap energy of 1.80 eV by a combined interaction of the crystal field and lattice strain. Then, the emission line observed at 1.87 eV in our samples may arise from the ^5^*E* → ^5^*T*_2_ radiative transitions. Hence, the band at 1.87 eV, could be appearing from the combined transitions involving – deep traped states and Mn^3+^ states.

**Figure 7:**
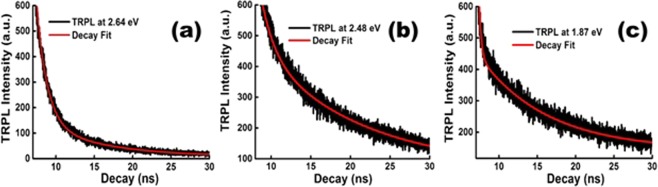
The lifetime decay plots of Mn doped CdSe nanosheets at PL energy (**a**) 2.64 eV, (**b**) 2.48 eV and (**c**) 1.87 eV. The red color spectrum is the second order exponential decay fitting curve.

In order to understand the electronic transitions quantitatively where the Mn^2+^ and Mn^3+^ atomic states coexist in the lattice, the lifetime of the transitions was estimated using time resolved photoluminescence (TRPL) spectroscopy. The TRPL spectra at PL lines 2.64 eV, 2.48 eV and 1.87 eV are shown in Fig. 7(a–c) respectively. Using a double exponential decay fitting, the lifetime and amplitude of the transitions were extracted and are listed in the Table 1. From the fit, the decay profile consists of relatively short component (1 to 3 ns) and long component (8 to 14 ns) lifetimes. The noteworthy features are the amplitude of the PL decay accompanied with highest probable radiative (short component) - and lowest probable nonradiative (long component) - transition with nearly similar order in lifetime. This type of the decay may be attributed to the *sp – d* exchange interaction between the charge carriers and localized Mn^2+^/Mn^3+^ magnetic dopant in the lattice. Here, the photo-induced magnetic coupling causes alignment of the localized spin in the volume of the exciton (excited carrieers bound states) and forms the excitonic magnetic polaron (EMP) even at zero magnetic field^51^. If the exciton lifetime faster than the EMP formation time, then, the assymety in spin orientation lead to finite probability of free exciton recombination over saturated EMP^52^. This corresponds to a double exponential decay accomplishing the short component and long componet transitions. In spite of long life bulk spin relaxation (in the order of micro-second), the nanosecond lifetime observed in our case is possible due to the strong admixture of the excitons corresponding to the magnetization (exchange field) associated excitonic Zeeman sublevels (overlapping of the bright exciton and dark exciton) in the layered nanostructures and confinement effect^53^. Again, the amplitude of the nonradiative decay corresponding to the Mn^2+^ is observed to be higher than in the case of Mn^3+^ PL line. This may be due to the degree of magnetic inhomogeneity in the respective EMP states. The above observations reveal the superior spin-active radiative transitions yielding optical properties in the entire visible range over Mn doped CdSe based quantum dots^20,40,54^, quantum well^20^ and nanoplatelates^41^.

**Table 1:** Life time decay parameters obtained from the dual decay fitting with experimental TRPL spectrum.

Energy (E)	τ_r_ (ns)	τ_nr_ (ns)	B_r_%	B_nr_%	Φ%
1.87	2.85	8.93	93.1	6.8	75.8
2.48	1.65	12.99	63.3	36.6	88.7
2.64	1.36	8.48	86.2	13.7	86.1

E indicates the PL energy. τ_r_, τ_nr_, are the lifetime (in nanosecond) and B_r_, B_nr_ are amplitude of the double exponential decay respectively. Φ refers to the quantum efficiency [Ф = τ_nr_/(τ_r_ + τ_nr_)].

**Figure 8:**
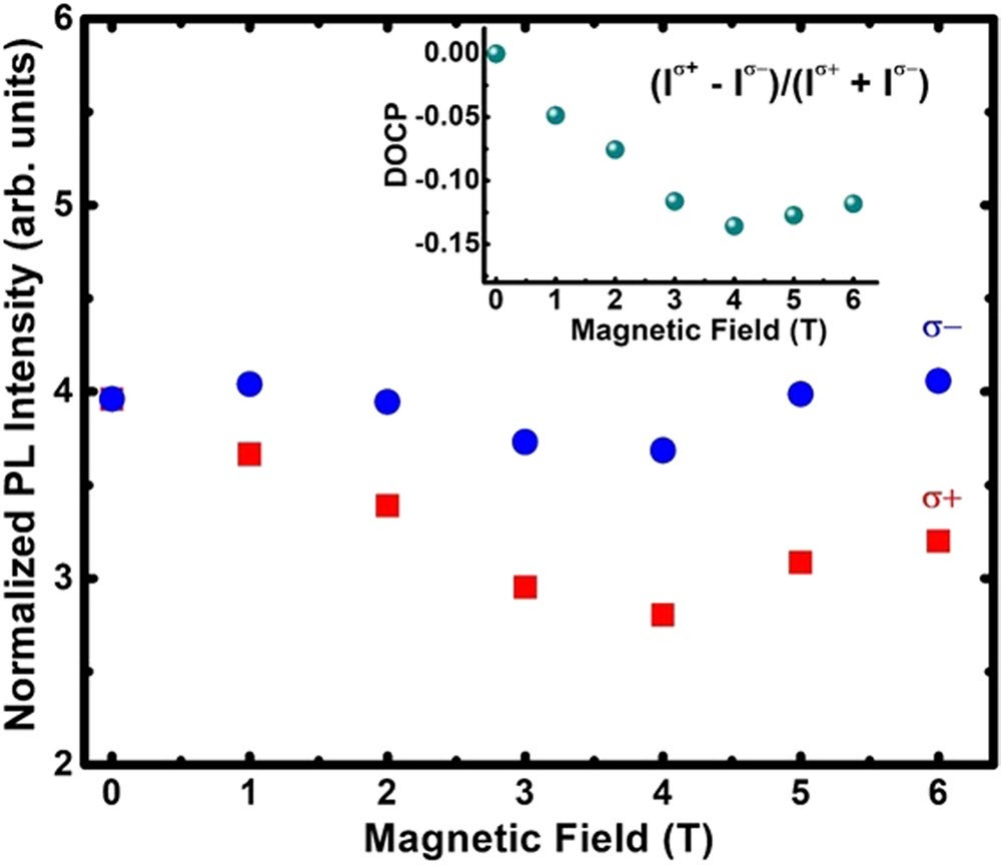
The normalized magneto-PL intensity versus magnetic field at temperature 2 K in *σ*^−^ and *σ*^+^ circular polarization condition represented by blue circles and red squares respectively. Inset is the plot of degree of circular polarization (DOCP) versus magnetic field.

Further, to corroborate the spin active optical properties of the (Cd,Mn)Se LNSs, the magnetic field (0–6 Tesla) dependent emission measurements were carried out at 2 K. A combination of a quarter-wave plate and a linear polaizer ensured resoltuion of two circular polarizations of the signal. As shown in Fig. 8, upon application of the magnetic field, the *σ*^−^ polarized emission is enhanced with respect to *σ*^+^ emission^41^. Inset to Fig. 8 shows the degree of circular polarization (*DOCP* $=[({I}^{\sigma +}-{I}^{\sigma -})/({I}^{\sigma +}+{I}^{\sigma -})]$) versus magnetic field. The absolute value of the *DOCP* increases with the increasing field and saturates at around 3T, as it was typically observed in case of bulk Mn-doped DMSs^8,55^. The total intensity, however, is roughly constant which remains in contrast with a typical behavior reported for Mn-doped DMSs. Previous studies of the (Cd,Mn)Se DMSs have shown that the excitonic signal gets stronger in *σ*^+^ polarization, while the sum of the intensity in both polarization increases in the magnetic field due to the deactivation of Mn-related quenching effect^56,57^. In parallel, the intra-ionic Mn^2+^ emission was reported to remain unpolarized in the magnetic field, and its total intensity was reported to decrease with the field.

Thus, in order to explain properties of magnetic field dependent emission in our study, we recall the recent work by R. Viswanatha *et al*.^58^ on small (Cd,Mn)Se nanocrystals, where comparable behavior as in our case reported. R. Viswanatha *et al*.^58^ have found *σ*^−^ polarized intra-ionic emission stronger than *σ*^+^ emission in magnetic field and explained this as a consequence of a particularly strong hybridization between Mn^2+^ and exciton states. Since the vertical dimension of our nanosheets are comparable to these nanocrystals, we conclude that the particularly strong hybridization between the Mn^2+^ and 2D exciton state is observed due to the closely located Mn^2+^ band and the bandgap of the (Cd,Mn)Se LNS. Therefore, the mixing of the Mn^2+^ wavefunction with that of the exciton confined within the (Cd,Mn)Se LNSs is likely to be responsible for dominating *σ*^−^ polarization of the emission in our work. This means that the observed emission comes from Mn ions intra-ionic transitions polarized due to a strong hybridization with excitons.

## Conclusion

In conclusion, the intriguing doping of Mn into the ultra-thin (thickness ~2.26 nm) CdSe layered nanosheets has been achieved and confirmed from the Raman scattering measurements. As observed from the XANES and EPR results, the dopant has been stabilized inside the CdSe LNS lattice in the form of multivalence of Mn^2+^ and Mn^3+^ states with an enhanced Lande-g factor (*g*_*eff*_ = 41.23). The interactions of localized *d*-electrons of Mn^2+^ and Mn^3+^ with CdSe LNS lattice depicts several magnetic phases leading to the low temperature ferromagnetic (transition temperature ~48 K) – and high temperature superparamagnetic – orderings. The magnetic field dependent circularly polarized PL measurements confirm the significance of spin polarized electronic states. The PL and TRPL analysis accomplished the spin active radiative transitions corresponding to NBE, Mn^2+^ and Mn^3+^ states yielding luminescence across the visible spectrum. The exceptional luminescence properties open up a way towards possible applications in large scale spin-active light emitting devices.
